# The Advances in Polymer-Based Electrothermal Composites: A Review

**DOI:** 10.3390/polym17152047

**Published:** 2025-07-27

**Authors:** Xiaoli Wu, Ting Yin, Wenyan Liu, Libo Wan, Yijun Liao

**Affiliations:** 1 School of Materials and Environmental Engineering, Chengdu Technological University, Chengdu 611730, China; wxli1@cdtu.edu.cn (X.W.);; 2 School of Humanities and Design, Chengdu Technological University, Chengdu 611730, China

**Keywords:** polymer-based electrothermal composites, electrothermal theoretical basis, electrothermal conversion efficiency, graphene, carbon nanotube, MXene

## Abstract

Polymer-based electrothermal composites (PECs) have been increasingly attracting attention in recent years owing to their flexibility, low density, and high electrothermal efficiency. However, although a large number of reviews have focused on flexible and transparent film heaters as well as polymer-based conductive composites, comprehensive reviews of polymer-based electrothermal composites remain limited. Herein, we provide a comprehensive review of recent advancements in polymer-based electrothermal materials. This review begins with an introduction to the electrothermal theoretical basis and the research progress of PECs incorporating various conductive fillers, such as graphene, carbon nanotubes (CNTs), carbon black (CB), MXenes, and metal nanowires. Furthermore, a critical discussion is provided to emphasize the factors influencing the electrothermal conversion efficiency of these composites. Meanwhile, the development of multi-functional electrothermal materials has been also summarized. Finally, the application progress, future prospects, limitations, and potential directions for PEC are discussed. This review aims to serve as a practical guide for engineers and researchers engaged in the development of polymer-based electrothermal composites.

## 1. Introduction

In recent years, with the development of nanotechnology, electrothermal technology has also developed rapidly and is widely applied in multiple industries [1,2]. The electrothermal performance of this technique relies on the Joule heat produced in the conductive materials. Traditional electrothermal materials include ceramic materials, metal alloys, and metal oxides, such as Ni-Cr (nichrome), Fe-Cr-Al, and indium tin oxide (ITO). However, these materials have disadvantages such as high density, high rigidity, complex manufacturing processes, low heating efficiency, and high cost [3]. Therefore, in recent years, researchers have focused on developing high-efficiency flexible electrothermal materials, including polymer-based electrothermal composites [4,5,6], film heaters fabricated via chemical vapor deposition (CVD) [7,8,9], and self-assembled film heaters [10]. Among them, polymer-based electrothermal composites have been increasingly drawing attention in recent years, owing to their advantages of low density, high toughness, low cost, ease of processing, and capability for mass production [4,11,12].

**Figure 1:**
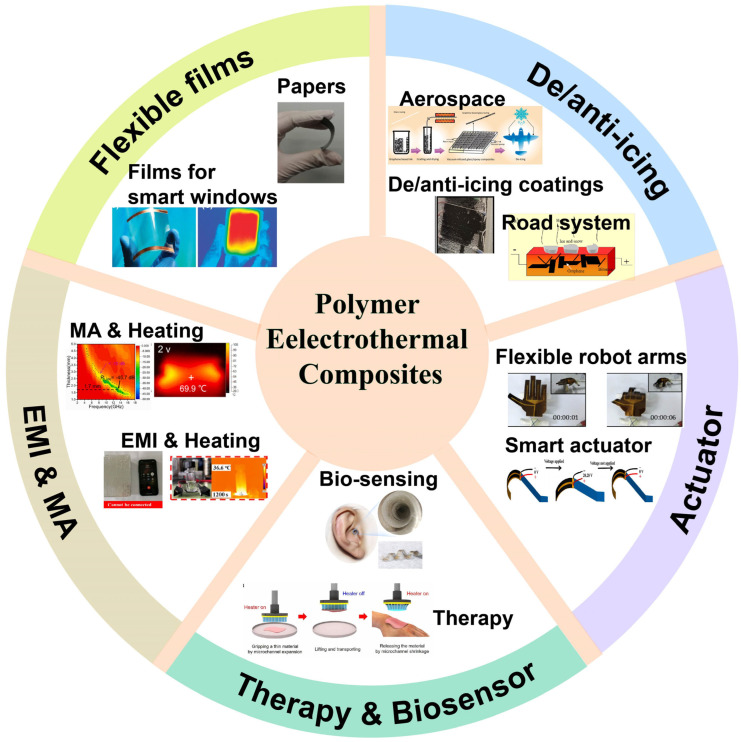
The applications of electric heating materials: de-icing/anti-icing [11,12,35], flexible films [6,44], electromagnetic interference (EMI) shielding [19] and microwave absorption (MA) [21], electric heating actuators [17,45], and physical therapy and medical monitoring [18,46].

Polymer-based electrothermal composites have been widely utilized across a broad range of applications, such as de-icing/anti-icing systems [11,13,14], household electric heating devices, anti-fogging systems [6], electrothermal actuators [15,16,17], physiotherapy devices [18], electromagnetic interference (EMI) shielding [19,20], and microwave absorption (MA) [21,22], as illustrated in Figure 1. These composites are generally fabricated by introducing conductive fillers into the polymer matrix [7]. Additionally, the electrical, electrothermal, and mechanical properties of polymer-based electrothermal composites can be modified to meet specific requirements by adjusting the concentration of conductive fillers [13].

**Figure 2:**
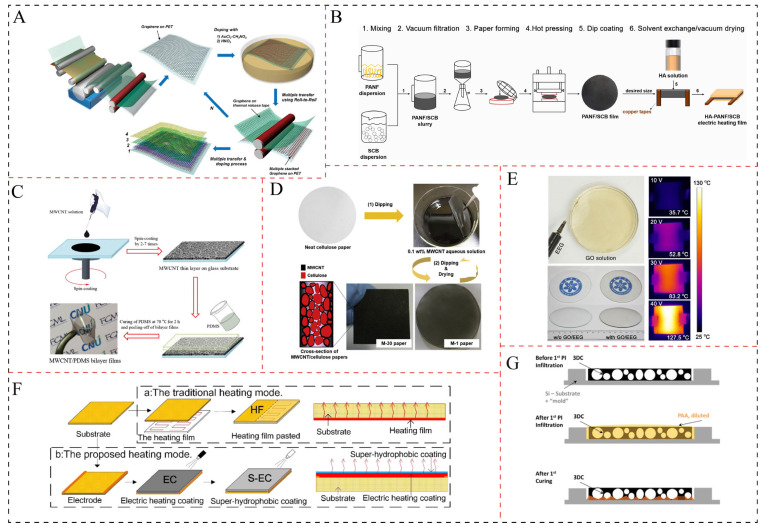
The preparation methods for polymer-based electrothermal materials: (**A**) CVD + roll–roll process [6], (**B**) solution mixing [24], (**C**) solution mixing + spin-coating [41], (**D**) dip-coating [34], (**E**) self-assembly process [10], (**F**) spray-coating [14], (**G**) CVD + infiltration process [43].

The conductive fillers primarily consist of carbon black (CB) [23,24], graphene [7,15,25], carbon nanotubes (CNTs) [26,27,28], Mxene [20], carbon fibers [29], silver nanowires [21], hybrid fillers [16,30,31], and others. Meanwhile, the polymer matrix predominantly includes polyethylene (PE), polyvinylidene fluoride (PVDF) [32], epoxy [4,33], cellulose [19,34], and asphalt [35], among others. Driven by the requirements of different scenarios, a variety of morphologies of electrothermal materials have been developed, such as flexible film [7,36], paper-like sheet [19,34], bulk structure [33], and coating layer [12,37,38]. The preparation methods for polymer-based electrothermal materials primarily encompass solution mixing [4,12,24], melt blending [35,39,40], self-assembly [10], spin-coating [41,42], spraying [14], infiltration [43], or combinations thereof, as illustrated in Figure 2.

While much of the focus has been intensifying on flexible and transparent film heaters and polymer-based conductive composites, comprehensive reviews of polymer-based electrothermal composites remain limited. Herein, we attempt to gather information regarding the progress concerning polymer-based electrothermal composites. Firstly, we briefly review the research progress of polymer electrothermal materials with different conductive fillers, which will enable us to understand their advantages and disadvantages. Furthermore, we discuss the influencing factors of the electrothermal conversion efficiency of electrothermal materials, which will help us understand the methods available to improve the electrothermal efficiency of electrothermal materials. We also review the application progress of electrothermal materials and further explore the future direction of polymer electrothermal materials, and discuss what needs to be done.

## 2. Theoretical Basis

The electrical conductivity and electrothermal performance of polymer-based electrothermal composites is generally obtained by the dispersion of conductive fillers within the polymer insulating matrix. Upon exceeding a specific threshold concentration of conductive fillers (percolation threshold, *p_c_*), an extensive network of conductive pathways forms within the matrix. These pathways enable the movement of free electrons along the conductive channels under an applied voltage, thereby facilitating current flow. In accordance with the Joule–Lenz law, heat is generated as current passes through the conductive fillers. The formula for the electrothermal conversion efficiency of these composite materials is presented as follows [7,16,47,48]:

(1) $$Q=I^{2}\cdot R\cdot t=P\cdot t$$

where *Q* represents the heat generation of the electric heating material; *I* is the current intensity flowing through the material; *R* is the resistance of the material; *t* is the heating time; and *P* is the power of the electric heating material. From the formula, it can be seen that the electric heating conversion efficiency is mainly determined by power. However, for the same power, the electric heating effect of materials with different areas varies greatly. Therefore, most researchers use power density to evaluate the electric heating conversion efficiency [9,16,49]. The formula is as follows:

(2) $$P_{d}=\frac{I^{2}R}{S\;}$$

where *P_d_* represents the heating power per unit area of the electric heating material; and *S* represents the working area of the electric heating material.

According to the above Joule heating formula, the reason for the generation of electrical heat conversion in electrothermal materials is that they have certain resistances. As reported in previous research works, the reasons for generating resistances mainly include two aspects. Firstly, the fillers are dispersed in the matrix to form conductive pathways. However, the conductive channels are not as unobstructed as those in metals. At the contact points of the fillers, there are Schottky barriers, and the hopping effect of charge carriers is relatively weak at the connection points, resulting in contact resistance [50,51,52,53].

At the same time, some fillers have interlayer barriers in the matrix, which are even larger. Secondly, the conductive fillers are not perfect. For example, graphene and carbon nanotubes both have various forms of defects, including morphological defects (such as pentagons and heptagons), voids, edges, cracks, and non-atomic impurities. Electrons are hindered to some extent by these defects, thus manifesting as resistance on a macroscopic scale. Microscopically, when an external voltage is applied to the conductive composite material, the electric field acts on the free charge carriers (free electrons or electron holes) within it. The charge carriers start to move along the conductive pathways in the conductive fillers. During the movement process, the charge carriers undergo inelastic collisions with the defects of the fillers, phonons, and connection points under the acceleration of the electric field, resulting in the conversion of kinetic energy into heat energy [54,55]. During the collision process, the collision frequency and the average energy of the charge carriers can be described. The collision frequency depends on the concentration of charge carriers and the density of conductive pathways, while the average energy of the charge carriers is related to the applied voltage [55]. When the voltage increases, the average energy of the charge carriers increases, the average free path of the charge carriers shortens, and more collisions occur, thereby generating more heat [56]. Therefore, the efficiency of electrical heat conversion in polymer-based electrothermal composite materials is related to the types and properties of conductive fillers, the dispersion structure of conductive fillers in the matrix, the carrier concentration of the composite material, and the applied voltage.

## 3. Research Progress of Polymer-Based Electrothermal Composites

### 3.1. Graphene Electrothermal Material

Graphene is a two-dimensional lattice composed of single-atom-thick layers, formed by carbon atoms with sp^2^ hybridization arranged in a honeycomb pattern in a dense manner [11]. It has a huge specific surface area (3000 m^2^/g) [57], superconductivity [58], high carrier mobility (over 10^6^ cm^2^·V^−1^·S^−1^) [59,60], extremely high mechanical properties [61], and excellent thermal conductivity (up to 5000 W/m·K [11]). Therefore, it is widely used for its conductive particles and introduced into polymers to prepare conductive and electrothermal composite materials. The high electrical conductivity and excellent thermal conductivity of graphene enable electrothermal composite materials to possess the advantages of rapid heating response, high electrothermal conversion efficiency, and uniform temperature distribution [6].

#### 3.1.1. Flexible Graphene/Polymer Bilayer Film Heater

**Figure 3:**
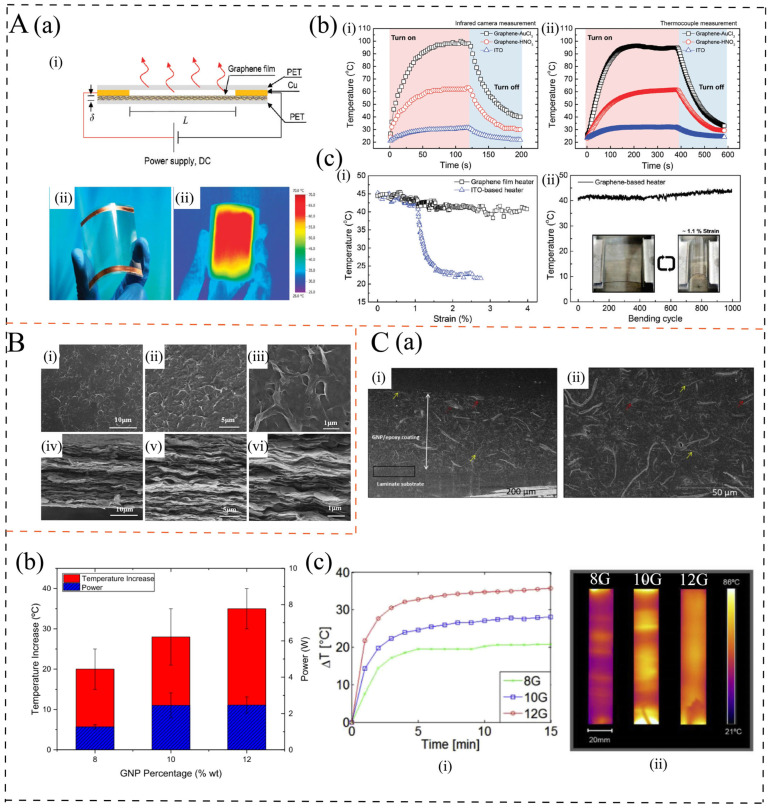
(**A**): (**a**) A schematic structure (i), an optical picture (ii), and infrared picture (iii) of a transparent, flexible graphene heater film [6]. (**b**) The temperature profiles of graphene-based heaters with two different doping agents and an ITO-based heater, measured by (i) an infrared scanner and (ii) a thermocouple (K-type) [6]. (**c**) Mechanical stability of the graphene-based heater including (i) variations in the temperature of a graphene-based heater compared with an ITO-based heater as a function of bending strain and (ii) mechanical stability test results of the graphene-based heater [6]. (**B**): The surface (i–iii) and cross-sectional (iv–vi) SEM images of the electrothermal films in different magnifications [36]. (**C**): (**a**) SEM images of epoxy coating doped with 10 wt% GNP over a laminate substrate, where (i) shows a lateral cross-section and (ii) shows the surface morphology [12]. (**b**) Electrical power and the maximum increase in temperature reached by the application of 800 V at room temperature [12]. (**c**) (i) Increase in temperature as a function of time and (ii) thermal images for increasing GNP wt.%, specifically 8 wt.% (8G), 10 wt.% (10G), and 12 wt.% (12G) [12].

In recent years, given the various limitations of ITO materials, including the rising cost of indium, complex fabrication processes, limited heating response, and high brittleness, flexible transparent electrothermal films based on graphene have attracted considerable attention from researchers. Since directly adding the filler to the polymer matrix to form a conductive network would affect the transparency of the film, many researchers have adopted a bilayer structure, attaching extremely thin graphene layers onto the transparent polymer matrix. The extremely thin graphene layers possess transparency as well as the effect of electric heating [62], thereby simultaneously achieving both transparency and electrothermal performance. Therefore, for these films, the preparation process of graphene films plays a critical role. The polymer matrix is generally polyethylene terephthalate (PET) due to its excellent transparency and flexibility [63]. Kang et al. [6] used a roll-to-roll method to transfer CVD-doped graphene onto PET to prepare a film heater. The film showed a high transmittance of 89% (Figure 3A(a)) and a low sheet resistance of 43 Ω/sq. It exhibited a high temperature increase of about 80 °C with a maximum heating rate of approximately 1.4 °C/s when a 12 V voltage is applied (Figure 3A(b)). Moreover, small changes in temperature deviation of approximately 1.02 °C were detected after the film was bent 1000 times with 1.1% strain (Figure 3A(c)). Due to the complexity and high cost of CVD and membrane transfer processes, some researchers have developed an easy and cost-effective method to fabricate a transparent flexible graphene-based film heater. Sun et al. [10] proposed a cost-effective self-assembly method to fabricate high-performance, large-area graphene oxide/electrochemically exfoliated graphene hybrid films for heater applications. The hybrid films exhibit a transmittance of 76.2%, and good heating characteristics and defogging performance. When a voltage of 40 V is applied for 60 s, these films achieve a saturation temperature as high as 127.5 °C.

In certain application fields, transparency is not essential. Therefore, many researchers have developed films with excellent flexibility properties. Li et al. [36] used a film transfer process to deposit pure graphene layers on PET to prepare a flexible electrothermal film. The steady-state temperature of the film reaches 139 °C, and the maximum heating rate is 13.7 °C/s. Sui et al. [3] fabricated a flexible rGO/polyimide (PI) bilayer film by solution mixing and a spin-coating process. The film could reach a steady-state temperature of 72 °C in less than 10 s with a maximum heating rate exceeding 16 °C/s at 60 V, and it showed pretty robust endurance against repeated bending. The excellent flexibility and electrothermal properties are attributed to the film structure, large surface areas, super-high electrical conductivity, and mechanical performance of graphene. As shown in Figure 3B [36], the graphene sheets are closely attached, forming a dense conductive network in the horizontal direction. Such graphene/polymer bilayer films exhibit excellent electrothermal performance; however, their preparation methods are relatively complex and costly, which limits their suitability for widespread application.

#### 3.1.2. Graphene/Polymer Electrothermal Composites

Electrothermal polymer-based composites with the incorporation of graphene into a polymer matrix are also gaining increasing attention due to their low cost, ease of processing, and capability for mass production. Compared with graphene/polymer bilayer films, their electrical heating performance is relatively poorer. This is because the content of graphene is lower and the formed conductive network is not dense enough, as shown in Figure 3C(a) [12]. They are generally fabricated using the solution mixing and melt blending method. For the graphene/polymer composites, the content of graphene is a key factor influencing the electrothermal performance. For instance, Redondo et al. [12] prepared GNP/epoxy resin coatings with graphene contents ranging from 8 to 12 wt.%, achieving electrical conductivity of 416-61 Ω·cm. The temperature increase was measured to be 20–35 °C, with heating rates increasing from 7.2 to 13.6 °C/min as the GNP content increased from 8 to 12 wt.%, as shown in Figure 3C. A similar result was obtained by Jeong et al. [4]. These results indicate that the electrothermal performance improves with increasing graphene content due to the increased electrical conductivity, see Table 1. However, higher content would decrease the maximum voltage the material can withstand [64,65]. In practical applications, low-voltage operation is often preferred. Nevertheless, in specific scenarios such as high-voltage transmission lines, high voltage operation is also necessary. For example, Wu et al. [66] fabricated a non-percolative rGO/XLPE composite where the fillers were not in direct contact for anti-icing/de-icing of transmission lines. This composite exhibited a high breakdown voltage of 1050–2600 V and demonstrated superior electrothermal behavior under high voltage due to the conduction taking place via field emission at high voltage.

From the above research, it can be observed that graphene/polymer bilayer films and graphene/polymer composites exhibit excellent electrothermal performance. However, they still have some limitations. For graphene/polymer bilayer films, the preparation process is complicated and the cost is high, and the thermal insulation polymer layer has low thermal conductivity, which affects heat transfer. For graphene/polymer composites, due to the large aspect ratio of graphene, it is easy to aggregate in the polymer matrix, which will affect the electrical conductivity and thus the power of electrothermal conversion and the uniformity of temperature distribution. A large number of researchers fabricated the composites using the solution mixing method to reduce the graphene aggregation, but this method is not suitable for large-scale production. Secondly, some researchers have modified the surface of graphene, but the electrical conductivity of graphene will be reduced as a result, and it is still unknown if the electrothermal stability will be affected after modification. Furthermore, thermal expansion will occur for the polymer matrix with the increase in temperature, causing the network established by graphene to break, thereby affecting the electrical conductivity and the power of electrothermal conversion. Although many researchers have focused on studying the temperature dependence of resistivity in graphene/polymer-based composites and the positive temperature coefficient (PTC) effect [67,68], the influence of PTC on electrothermal performance has not been thoroughly and systematically investigated. Therefore, in the future, researchers still need to conduct further studies on these aspects to improve the electrothermal stability of graphene/polymer composites and ensure the reliability of practical applications.

### 3.2. Carbon Nanotube Electrothermal Composites

Carbon nanotube (CNT)-based electrothermal polymer composites have become a research hotspot in recent years [69,70], as carbon nanotubes (CNTs) possess an extremely high aspect ratio (typically ranging from 10^2^ to 10^5^) [71], excellent electrical conductivity (typically 1000–2000 S/cm) [72], high thermal conductivity, superior mechanical properties [73], remarkable flexibility [74], and ultrahigh thermal stability. Moreover, owing to the unique quasi-one-dimensional nanostructure of CNTs, electrothermal composites with aligned carbon nanotubes have also garnered significant attention. Currently, CNT-based electrothermal materials encompass flexible CNT film heaters [8,75,76] as well as highly oriented CNT-based electrothermal films [77,78].

#### 3.2.1. Flexible CNT/Polymer Bilayer Film Heater

**Figure 4:**
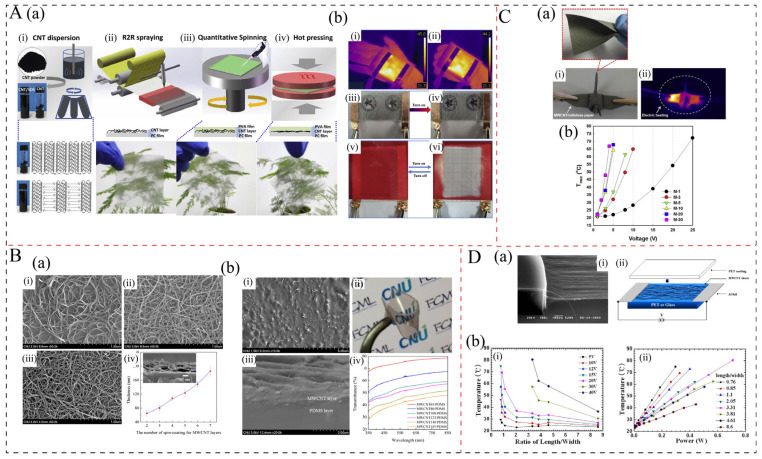
(**A**): (**a**) Schematic illustrations of the self-developed fabrication process for PVA/CNTs film: (i) Schematic diagram of CNTs dispersion and corresponding optical image; (ii) Spraying method for pre-constructing CNT network on polycarbonate surface; (iii) quantitative spinning process to introduce ultrathin PVA layer; (iv) hot pressing treatment to prepare PVA/CNTs film [42]. (**b**) (i,ii) Thermal images of PVA/CNTs heater affixed on hand in different states; (iii,iv) the defrosting result of PVA/CNTs heater before and after the heating process; (v, vi) reversible thermochromism is also observed in a dyed film. [42]. (**B**): (**a**) (i–iii) SEM images of MWCNT layers prepared by different cycle numbers (2, 4, and 6) of spin-coating on glass plates; (iv) change of average thickness of MWCNT layers as a function of the cycle number of spin-coating and a typical SEM image of cross-section of MWCNT layer formed by 7 cycles of spin-coating on a glass plate [41]. (**b**) SEM images of surface (i) and cross-section (ii) of MWCNT108/PDMS bilayer film with 108 nm thickness of the MWCNT layers; (iii) optical image of MWCNT108/PDMS bilayer film; (iv) transmittance of MWCNT/PDMS bilayer films with different MWCNT layer thicknesses [41]. (**C**): (**a**) (i) Typical digital and (ii) infrared images for the paper crane made of M-1 paper under an applied voltage (M-x, where x denotes the cycle number of dip-coating processes) [34]. (**b**) Changes in steady-state maximum temperature (T_max_) of MWCNT/cellulose papers as a function of the applied voltage [34]. (**D**): (**a**) (i) Scanning electron microscopy (SEM) image of MWCNT sheets from well-aligned MWCNTs on a substrate and (ii) the diagram of MWCNT sheet film on a substrate with an electrode pair [76]. (**b**) (i) Sheet temperatures plotted versus length/width at different DC voltages, and (ii) sheet temperature plotted versus applied DC power to sheet film for each length-to-width ratio [76].

The flexible CNT/polymer bilayer film heater is fabricated by depositing a pure CNT layer onto the surface of a polymer substrate, which is similar to a graphene/polymer bilayer film. This is also due to the high transparency, low resistance, and excellent mechanical flexibility of CNTs [79,80]. These films are mainly prepared by solution mixing combined with techniques such as dry and wet spin-coating [75], dip-coating [34], spray-coating [81], and printing [82]. The main polymer matrices are PET, polydimethylsiloxane (PDMS), and poly(vinyl alcohol) (PVA). Zhou et al. [42] fabricated ultrathin flexible poly(vinyl alcohol) (PVA)/CNT transparent film heaters (TFHs) with a fast response time using a green all-water-based solution process (Figure 4A(a)). The resulting film shows good optical and electrical properties of 475 Ω/aq with a transmittance of 77.3%, and reveals a high heating temperature increase (Figure 4A(b)), a very fast heating time, and rate of 90 °C, 8 s, and 11.4 °C/s, respectively, under 15 V. Compared with two-dimensional graphene, carbon nanotubes (CNTs), although being a one-dimensional structure, possess an extremely large aspect ratio and are capable of forming a dense, interwoven, and continuous network, as illustrated in Figure 4B(a). This structural characteristic accounts for the high electrothermal conversion efficiency observed in such CNT-based films. However, the thickness and dense continuous networks can affect the transparency of the film. For example, Yan et al. [41] prepared 65–185 nm MWCNT films on PDMS by the spin-coating method. They found that as the film thickness increased, the transparency decreased, as shown in Figure 4B(b).

#### 3.2.2. CNT/Polymer Electrothermal Composites

CNT/polymer electrothermal composites with the incorporation of CNTs into the polymer matrix also exhibit remarkable electrothermal performance; however, their transparency is poorer than that of CNT/polymer bilayer film. They are generally fabricated using solution mixing methods combined with dip-coating [34] and spray-coating [81] techniques, which have the ability to reduce the aggregation or entanglement of CNTs resulting from the one-dimensional structure and high aspect ratio of CNTs. Lee et al. [34] developed a series of multi-walled carbon nanotube (MWCNT)-coated cellulose papers via a simple dip-coating process. The results demonstrated that with an increase in the dip-coating cycle from 1 to 30, the CNT content increased from 1.5 wt.% to 13.3 wt.%, while the in-plane electrical conductivity increased from 0.02 S/cm to 1.11 S/cm. Additionally, the papers showed high flexibility and excellent electrothermal performance, as shown in Figure 4C. At a CNT loading of 1.5 wt.% and 13.3 wt.%, the temperature increased by approximately 50 °C under 25 V and 45 °C at 3 V, respectively. Lu et al. [19] prepared MWCNT/cellulose composites with MWCNT contents ranging from 10 to 50 wt.% through the solution mixing process. Their electrical conductivity varied from 1.4 S/cm to 19.0 S/cm. At MWCNT loadings of 10, 30, and 50 wt.%, the composites achieved heating temperatures of 30 °C, 100 °C, and 146 °C, respectively, under a low voltage of 2 V, with corresponding heating rates of 0.15 °C/s, 3.33 °C/s, and 4.86 °C/s. It can be seen from these studies that the electrothermal performance of CNT/polymer composites is mostly poorer than that of CNT/polymer bilayer films, which should be related to the lower content resulting in a lower electrical conductivity.

#### 3.2.3. Aligned CNT/Polymer Bilayer Film Heater

For aligned CNT/polymer electrothermal films, they are generally fabricated by CVD techniques and spinning processes to deposit aligned CNTs onto PET substrates [49,76], as illustrated in Figure 4D(a) [76]. Although these films demonstrate superior electrothermal properties, their fabrication process is relatively complex and cannot be mass-produced. Furthermore, the oriented CNT films exhibit anisotropy characteristics, which limit their application in certain fields. Additionally, their electrothermal properties are influenced by many factors. First, the choice of substrate plays a critical role; for instance, Janas et al. [75] fabricated substrate-free aligned CNT films using CVD and spinning techniques, achieving an electrical conductivity of 10^2^ S/cm and enabling the heating temperature to increase from room temperature to 410 °C within 0.5 s. This performance was notably superior to that of films with substrates due to the slower heat-transfer coefficient associated with the substrate material. Second, the thickness of the CNT layer also affects the electrothermal performance; thicker layers generally exhibit higher electrical conductivity and enhanced electrothermal performance. Moreover, the quality of CNTs, including their purity, alignment, and aspect ratio (Figure 4D(b) [76]), which are dependent on the CVD process parameters, can significantly influence electrothermal performance.

As indicated in the aforementioned research, both CNTs and graphene serve as primary conductive particles in polymer-based composites and can be utilized to fabricate transparent flexible film heaters and composites with superior electrothermal performance. However, compared to graphene, CNTs exhibit slightly lower electrical and thermal conductivities. Additionally, their extremely high aspect ratio leads to a tendency for entanglement and aggregation during dispersion in polymers, which results in poorer uniformity and electrothermal properties compared to graphene-based composites. To address the dispersion problems of CNTs, many researchers have employed acid treatments and surfactants [6]; however, these methods may also influence the intrinsic electrical conductivity and electrothermal performance of CNTs.

Therefore, enhancing the dispersion of CNTs without compromising their electrical conductivity and thereby improving the overall electrothermal performance remains a critical direction for future research.

### 3.3. Carbon Black Electrothermal Composites

**Figure 5:**
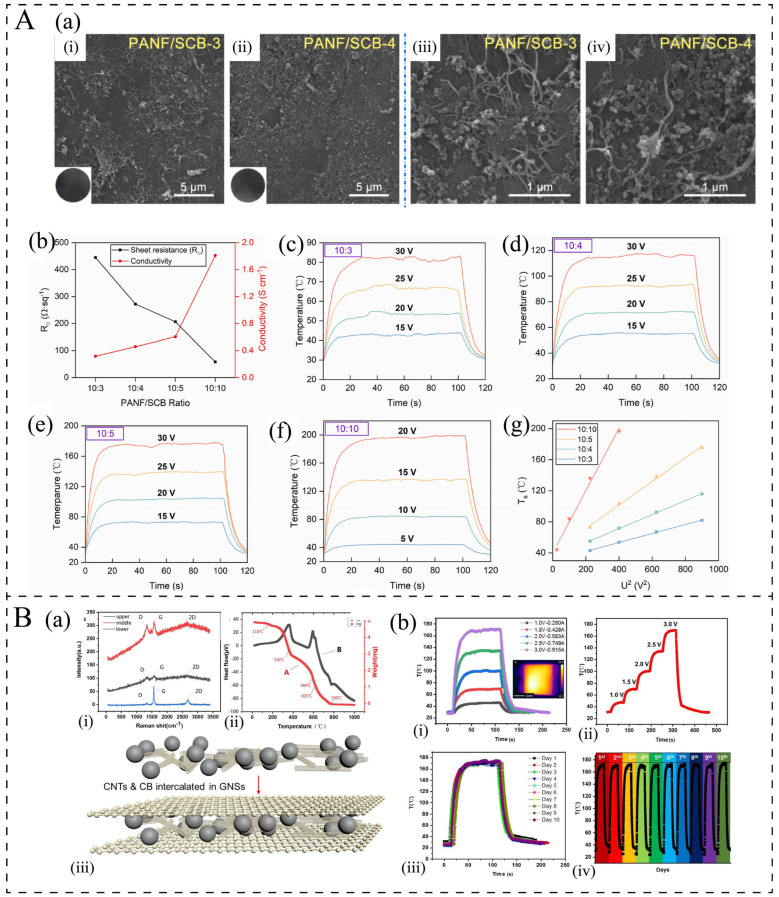
(**A**): (**a**) SEM images of the surface of PANF/SCB films with 23 and 28.5 wt.% SCBs at ×3000 (i,ii) and ×20000 (iii,iv) magnification, respectively. The inset shows digital images of PANF/SCB films [24]. (**b**–**g**) Electric heating performance of HA-PANF/SCB films with different PANF/SCB mass ratios [24]. (**B**): (**a**) (i) Raman spectrogram of GNSs in different layers, (ii) Thermal gravimetric analysis and Differential Thermal Analysis results of 3D intercalation GNS/MWCNT/CB composite, and (iii) schematic diagram of CNTs and CB intercalated in GNS [87]. (**b**) (i) Electrothermal performance of the flexible electrothermal film under different voltages; (ii) performance of the flexible electrothermal film with continuously changing voltage; (iii, iv) performance and the stability test of the flexible electrothermal film in air [87].

Superconducting carbon black (SCB) is a high-structured carbon black that is well suited for anti-static and conductive applications [83,84]. Carbon black typically exhibits a spherical morphology; however, it tends to aggregate into small clusters with minor branching structures, which facilitates the formation of conductive networks within the polymer matrix [85]. Furthermore, compared to costly carbon nanotubes and graphene, SCB exhibits significantly lower production costs and is amenable to large-scale preparation [86]. Consequently, SCB can be considered an effective conductive filler for fabricating carbon-based polymer electrothermal materials. However, due to its significantly lower electrical conductivity and aspect ratio—in that it is more difficult for the particles to come into contact with each other and form a conductive network, as illustrated in Figure 5A(a)—compared to graphene and carbon nanotubes, a higher loading of SCB is required to achieve comparable electric heating performance, see Table 1. As illustrated in Figure 5A, Chen et al. [24] fabricated PANF/SCB electric heating films with SCB contents ranging from 23 to 50 wt.% using the solution mixing method, followed by coating with a layer of heterocyclic aramid (HA). The conductivity of the HA-PANF/SCB films was measured at 0.3–1.8 S/cm. At an SCB content of 23 wt.%, the film exhibited a temperature increase of approximately 50 °C under a voltage of 20 V; at 50 wt.% SCB content, the temperature increased by 43.9 °C at 5 V and 196.4 °C at 20 V. Despite their high electrothermal performance, higher filler loadings deteriorate the mechanical properties of the composite.

Due to these limitations, the number of studies of the electrothermal properties of CB/polymer composites has been significantly lower than those of graphene and CNTs in the past 10 years. Most of the studies have involved adding SCB as a second or third phase to electrothermal materials. Its spherical nanostructure can fill the gaps in the conductive network, enhancing the conductivity and the penetration threshold, and it can greatly reduce the production cost [30,87,88]. As shown in Figure 5B(a), Liao et al. [87] fabricated a novel 3D intercalation graphene nanosheet (GNS)/MWCNT/CB composite. They found that the milled nanoscale CB was filled into an acid-treated-MWCNT network and intercalated in GNSs. This reduced the seepage threshold and formed a more uniform 3D conductive path (4 Ω sq^−1^). The composite exhibited excellent electrothermal radiation conversion efficiency (74.75%) and could reach a high temperature (175 °C) under a low voltage (3.0 V) (Figure 5B(b)). Moreover, the mechanical properties of the flexible electrothermal films are outstanding. Similar results have been obtained by Wang et al. [30] and Huang et.al. [88]. Therefore, adding SCB as the second or third phase to the polymer matrix to obtain an electrothermal material is a preferable option.

### 3.4. Mxene Electrothermal Composites

**Figure 6:**
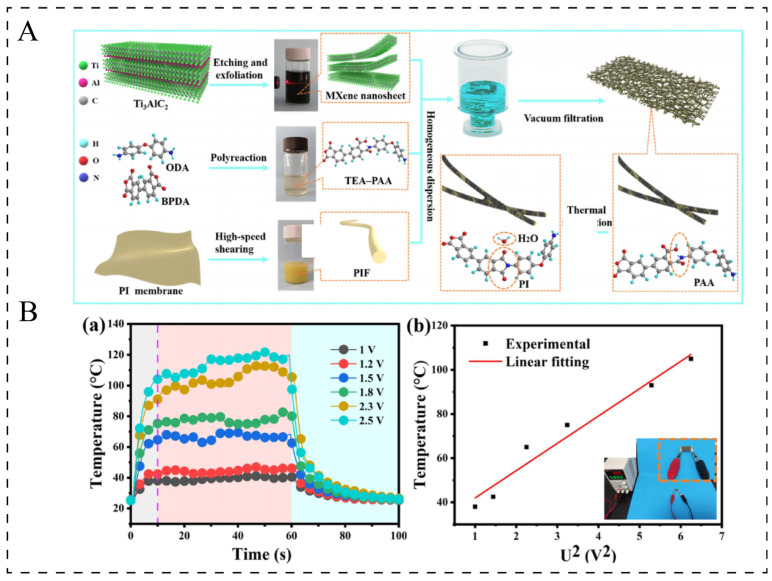
(**A**): Schematic illustration for the fabrication of flexible conductive PIF/MXene composite films [20]. (**B**): (**a**) Time-dependent surface temperature profile of PM-49.1 under different voltages [20]. (**b**) Experimental data and linear fitting of saturated temperature vs. U^2^ [20].

Recently, Ti_3_C_2_T_x_ MXene, as part of the newly developed two-dimensional transition-metal carbides and/or nitrides, has garnered significant attention for its potential in thermal management applications. This is due to its exceptional metallic-like conductivity (reaching up to 5225 S·cm^−1^) [89], substantial specific surface area, and outstanding mechanical properties [90]. Gong et al. [91] developed a composite film consisting of phase change capsules, MXene, and polyvinyl alcohol using MXene mortar. This material enabled Joule heating when stimulated by a low voltage of 1.5 V. Additionally, Xie et al. [92] introduced a high-performance, flexible piezoresistive sensor based on a 3D MXene/polyethylenimine network. This sensor not only monitored various human activities in real-time but also demonstrated remarkable Joule heating capabilities. Sun et al. [20] fabricated a flexible conductive polyimide fiber (PIF)/MXene composite film using the solution blending method (Figure 6A). When the content was 49.1 wt.%, the electrical conductivity was as high as 37.88 S/cm, with a maximum temperature rise of 80 °C at 2.5 V and a maximum heating rate of 8 °C/s (Figure 6B). Zhou et al. [93] prepared alternating multilayered cellulose nanofiber (CNF)@MXene films using an alternating vacuum filtration (AVF) process. The electrical conductivity was 6.21–0.82 S/cm, with a maximum temperature rise of 92 °C at 6 V and a heating rate of 9.2 °C/s.

The above studies show that MXene/polymer composite materials possess excellent electrothermal property. However, compared with carbon-based electrothermal materials, MXene—as a novel type of conductive particle—still exhibits several limitations, including a complex and potentially hazardous synthesis process, high production costs, being currently limited to the experimental stage, and the inability to achieve large-scale manufacturing. Moreover, MXene is susceptible to oxidation and degradation, and its nanosheets tend to agglomerate, which compromises electrical conductivity [94]. Therefore, if MXene can make progress in green synthesis, large-scale production, and stability optimization, MXene and its composite materials are expected to achieve commercial application in specific high-value-added fields.

### 3.5. Metal Nanowire Electrothermal Composites

**Figure 7:**
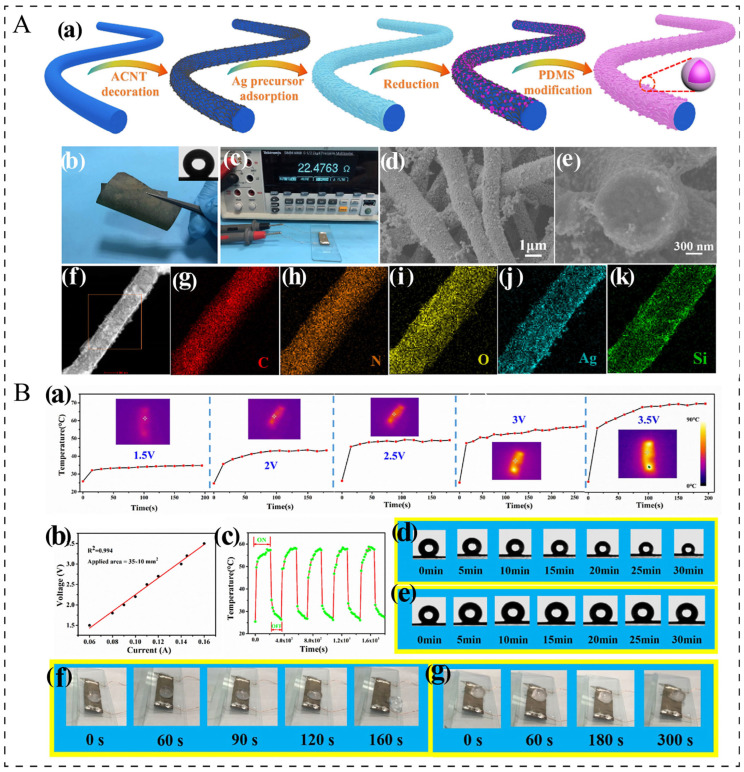
(**A**): (**a**)Schematic illustration of the preparation process of superhydrophobic and electrically conductive TPU/ACNT/AgNP/PDMS nanofiber composites [95]. (**b**) Photograph of the CNC membrane with good flexibility [95]. (**c**) Photo showing the resistance of one piece of CNC membrane [95]. SEM image for (**d**) surface and (**e**) cross-sectional morphology [95]. (**f**) TEM image of the CNC membrane [95]. (**g**–**k**) Element mapping images for C, N, O, Ag, and Si, respectively [95]. (**B**): (**a**) Temperature variation of the CNC at different applied voltages. (**b**) Voltage–current curve of the CNC [95]. (**c**) Temperature variation of the CNC experiencing cyclic heating–cooling process at a given voltage of 3 V [95]. Water droplet on the CNC surface (**d**) with a voltage of 3 V and (**e**) without an applied voltage [95]. De-icing performance of the CNC (**f**) at a voltage of 3 V and (**g**) without an applied voltage [95].

Due to their excellent intrinsic electrical conductivity, full-spectrum transparency, and outstanding mechanical strength, metal nanowire networks (MNNs), especially silver nanowire (AgNW) and copper nanowire (CuNW) networks, are regarded as highly promising substitutes for ITO in next-generation electronic devices and heaters. However, in the majority of cases, MNNs are incorporated into composite materials either by being combined with other conductive fillers or through a coating process. For instance, Lin et al. [95] developed conductive nanofiber composites (CNCs) through the sequential deposition of acid-modified carbon nanotubes (ACNTs), silver nanoparticles (AgNPs), and polydimethylsiloxane (PDMS) onto the surface of elastic thermoplastic polyurethane (TPU) nanofibers (Figure 7A). These multi-functional CNCs exhibit high conductivity (reaching up to 30.32 S/cm) and superhydrophobic performance (Figure 7B), leading to superior electrothermal properties and efficient de-icing capabilities. At 3.5 V, the temperature of composite increases by 44.6 °C, with the maximum heating rate being approximately 1.0 °C/s. Han et al. [21] fabricated carbon cloth (CC)@ZnO/silver nanowire (AgNW)/polyvinyl alcohol (PVA) composites through a typical hydrothermal method and layer-by-layer coating method. These films demonstrated exceptional Joule heating capabilities even at low voltages. Specifically, when subjected to 2 V, the temperature increased by 44.9 °C. Despite their high electrical conductivity and superior electrothermal properties, metal nanowires are not ideal as conductive additives for electrothermal applications, compared to carbon nanoparticles and MXene, due to their limited oxidation resistance and thermal instability [96].

**Table 1:** The electrothermal performance of polymer-based electrothermal composites.

Material	Method	*ψ/* wt.%	Thickness/μm	*ρ*/Ω cm	*V*/V	*P_d_*/ W/cm^2^	*r/*(°C/s)	Δ*T*/°C	Ref.
GNP film-PET	CVD + roll-to-roll	100	n.a.	43 Ω/sq	12	0.213	1.33	80	[6]
GNP film-PET	Film transfer	100	n.a.	159 Ω/sq	30	0.113	13.7	139	[36]
GNP/epoxy film	Solution mixing	2	100	10^2^–10^4^	100	0.1	1.6	40	[4]
		5			70	0.25	3.6	90	
		10			30	0.5	4.2	106	
Graphene Nanoribbon/epoxy	Solution mixing	5	5000	<1	40	0.5	n.a.	160	[33]
GNP/epoxy coating	Solution mixing	8	194	61–416	800	0.2	0.12	20	[12]
		10				0.3	0.17	28	
		12				0.4	0.23	30	
MWCNT Films-PDMS	Spin-coating	100	0.065	10^5^–10^3^ Ω/sq	100	0.07	n.a.	40	[41]
			0.123		90	0.56	n.a.	215	
			0.183		60	0.42	n.a.	180	
SWCNT films	Spray- coating	100	n.a.	130–190 Ω/sq	60	n.a.	n.a.	135	[81]
MWCNTs/cellulose papers	Dip-coating	1.5	157.3–170.1	0.9–50	25	n.a.	1	50	[34]
		13.3			3	n.a.	1	45	
MCNTs/cellulose film	Solution mixing	10	300	0.05–0.7	2	0.1	1	30	[19]
		30				0.3	3.33	100	
		50				1	4.86	146	
Aligned CNT unsupported films	CVD	100	n.a.	10^−2^	n.a.	3.33	750	375	[75]
Aligned CNT film-PET	CVD	100	0.2	699 Ω/sq		0.542	1.0	52	[76]
Aligned CNT film/GF/epoxy	CVD	n.a.	24	20.9 Ω/sq	16	0.49	2.6	140	[78]
CB/PVDF	Solution mixing	7	n.a.	10^0^–10^4^	36	0.075	n.a.	8	[32]
		10				0.15	n.a.	15	
		7			60	0.3	n.a.	40	
		10				0.55	0.4	60	
SCB/PANF	Solution mixing	23	76–122	0.3~1.8	20	n.a.	n.a.	20	[24]
		33				n.a.	n.a.	140	
		50				n.a.	n.a.	196	
PIF/MXene film	Solution mixing	43.1	132	0.026	2.5	n.a.	10.5	105	[20]
Alternating multilayered CNF@MXene films	Alternating vacuum filtration	100	35	621–82	3	n.a.	5.8	58	[93]
					6	n.a.	11.4	114	
CC@ZnO/AgNWs/PVA films	Layer-by-layer coating	n.a.	n.a.	n.a.	2	n.a.	0.9	45	[21]

where *ψ, ρ*, *V, P_d_, r,* Δ*T* are the content of fillers, electrical resistivity, applied voltage, power density, heating rate, and temperature increase values.

## 4. Factors Affecting Electric Heating Performance

### 4.1. Electrical Conductivity

Based on the Joule heating formula P = U^2^/R, it is evident that the efficiency of electric heating conversion is mainly determined by the resistance of the composite. The resistance is influenced by factors such as the electrical conductivity of the composite, and the size and thickness of the sample, among others. Among these factors, electrical conductivity plays a crucial role. It is influenced by the content of conductive fillers, their intrinsic electrical conductivity, shape, aspect ratio, and surface area, as well as their dispersion and orientation within the matrix. Additionally, it is affected by the fabrication process and the properties of the polymer matrix. Table 2 summarizes the electrical conductivity values for polymer-based composites with different types of conductive particle, matrix, and preparation method. Raji et al. [33] fabricated an electric heating composite by incorporating graphene nanoribbons into epoxy resin. The percolation threshold was approximately 1–2 wt.%, and at a loading of 5 wt.%, the electrical conductivity exceeded 1 S/cm, enabling a temperature increase of approximately 90 °C under a voltage of 30 V. Both the electrical conductivity and heating performance were significantly higher than those of the graphene/epoxy film prepared by Jeong et al. [4]. Furthermore, experimental and simulation studies have demonstrated that fillers with a higher aspect ratio are more likely to form conductive networks and exhibit lower percolation thresholds. [97] However, some researchers report that two-dimensional nanosheets, such as graphene nanosheets, have superior performance due to their high specific surface area and ease of modification with various molecules [98]. Additionally, most studies indicate that among several mixing methods, solution mixing enables the composites to possess the highest electrical conductivity. Melt blending often results in particle re-aggregation and wear, reducing the lateral dimensions of graphene. In situ polymerization forms covalent bonds between the matrix and fillers, which can hinder direct contact between fillers and decrease the effective aspect ratio [99,100,101].

The influence of the matrix on the conductivity is also significant. Research has shown that when the matrix viscosity is low, at the same content, the conductivity of resin with lower viscosity is higher than that of resin with higher viscosity. This is because resin with lower viscosity is more uniformly mixed with the fillers during the mixing process, which is conducive to the establishment of the conductive network [102]. According to the Klason theory [103], an increase in the crystallinity of the matrix enhances the conductivity of the composite material. At room temperature, conductive fillers are unable to penetrate the crystalline regions and are instead confined to the amorphous regions or interphase boundaries, where they form conductive networks. Conversely, some studies indicate that semi-crystalline matrices, such as PP, PE, PEO, and PA, exhibit higher percolation thresholds and conductivities compared to non-crystalline matrices like epoxy and silicone rubber. This is because the semi-crystalline matrix induces phase separation during crystallization, causing the fillers to be expelled into the amorphous regions, which significantly affects the uniformity of filler dispersion [102,104].

**Table 2:** The electrical conductivity of polymer-based composites.

Polymer	Fillers	Method	*p_c_*	*ρ*/(S/cm)	Ref.
PMMA	rGO	Solution mixing	0.25 vol.%	~10^−4^	[105]
P(MMA-co-BA)	GNP	Emulsion polymerization	0.1 vol.%	2.17	[106]
PMMA	GNP	Solution mixing	0.4 vol.%	~10^−3^	[107]
PS	GNP	Solution mixing	0.33 vol.%	0.0349	[108]
	CNT		0.5 vol.% (1.08 wt.%)	~10^−3^	
PS/PLA	GNP		0.075 vol.%	~10^−3^	
TPU	rGO	Solution mixing	<0.3	n.a.	[101]
		In situ polymerization	0.3–0.5		
		Melt blending	1–1.5		
Epoxy	GNP	Solution mixing	0.5–1 vol.%	10^−4^	[109]
Epoxy	CNT	Solution mixing	0.5–1 vol.%		[110]
PP	CNT	Melt blending	7.5 wt.%	~10^−8^	[111]
	rGO		5 wt.%	~10^−6^	
	CB		7.5 wt.%	~10^−7^	
LLDPE	rGO	Melt blending	0.5–0.9 vol.%	~10^−4^	[112]
HDPE	CNT	Powder dispersion and hot-pressing process	0.15 vol.% (0.3 wt.%)	~10^−2^	[113]
	GNP		0.953 vol.%	~10^−5^	
PP	MWCNT	Melt blending	0.22 wt.%	10^−3^	[114]
PP	S-MWCNT	Melt blending	>1 wt.%	n.a.	[115]
	L-MWCNT		0.1 wt.%	n.a.	
Epoxy	CNT	Solution mixing	0.0025 wt.%	0.01	[83]
	CCB		1 wt.%	10^−3^	

### 4.2. The Size of Heaters

**Figure 8:**
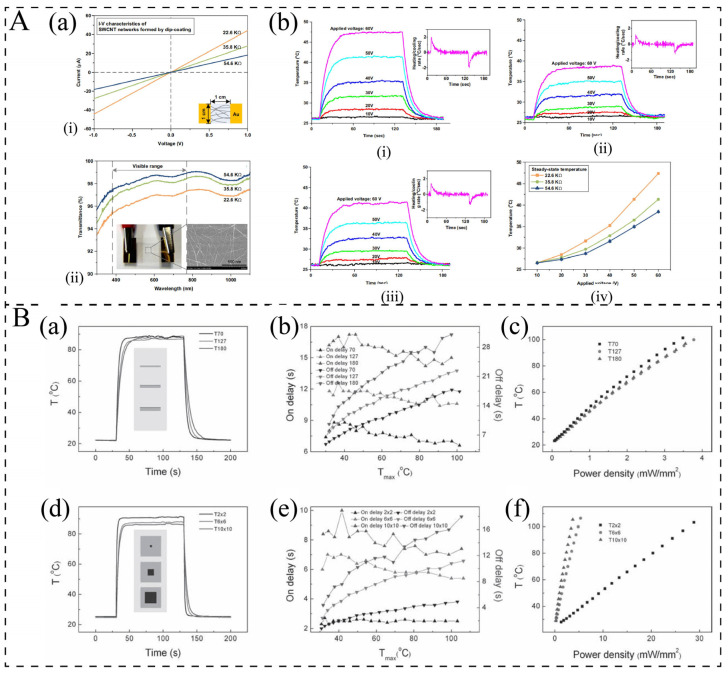
(**A**): (**a**) (i) I-V measurement and (ii) transmittance of SWCNT-coated glass specimens [116]. (**b**) (i–iii) Temperature profiles of the SWCNT-coated specimen with resistances of 22.6, 35.8, and 54.6 KΩ, respectively, with respect to the applied voltage. The plot of the derivative of the temperature vs. time is shown in the inset at the applied voltage of 60 V; (iv) average temperature of the specimens at the steady-state with respect to the applied voltage [116]. (**B**): (**a**) Response to the heating signal, (**b**) on and off delays, and (**c**) power-density–temperature plots of the CNT film on PET for different substrate thicknesses of 70, 127, and 180 μm [49]. (**d**) Response to the heating signal, (**e**) on and off delays, and (**f**) power-density–temperature plots of the CNT film on PET for different CNT film areas of 2 × 2, 6 × 6, and 10 × 10 mm^2^ [49].

In addition to the electrical conductivity of composite materials, the dimensions of the sample, including length, width, and thickness, also play a critical role in influencing electrothermal properties. Kang et al. [116] investigated the influence of film thickness on electrothermal performance by controlling the film thickness through different impregnation times on a glass substrate. The results indicated that as the thickness increased, the resistance decreased, leading to a higher temperature increase with increasing thickness (Figure 8A). However, the transmittance decreased as the thickness increased. Based on the formula P = U^2^/R, when the resistance doubles, the power doubles as well. However, the resistance difference was 2 times but the temperature increase value was significantly lower than 2 times, which is related to the density and porosity of the material causing heat loss and the increase in heat convection due to the reduction in resistance.

Liu et al. [49] fabricated a super-aligned carbon nanotube (CNT) film heater on a polyethylene terephthalate (PET) substrate. They observed that thinner substrates resulted in faster thermal responses (Figure 8B(a,b)), and the thickness of PET does not cause obvious differences in power consumption (Figure 8B(c)). Furthermore, the heating area not only affects the resistance but also the convective heat-transfer coefficient. The heat-transfer coefficient increases inversely with the increase in sample area, thereby enhancing the thermal response of the film heater [49,117] (Figure 8B(d,e)). However, for a given temperature, the power density increases with a decrease in the heating area and an increase in the heat-transfer coefficient, as a result of enhanced heat dissipation [117,118], as shown in Figure 8B(f).

### 4.3. Heat Dissipation

**Figure 9:**
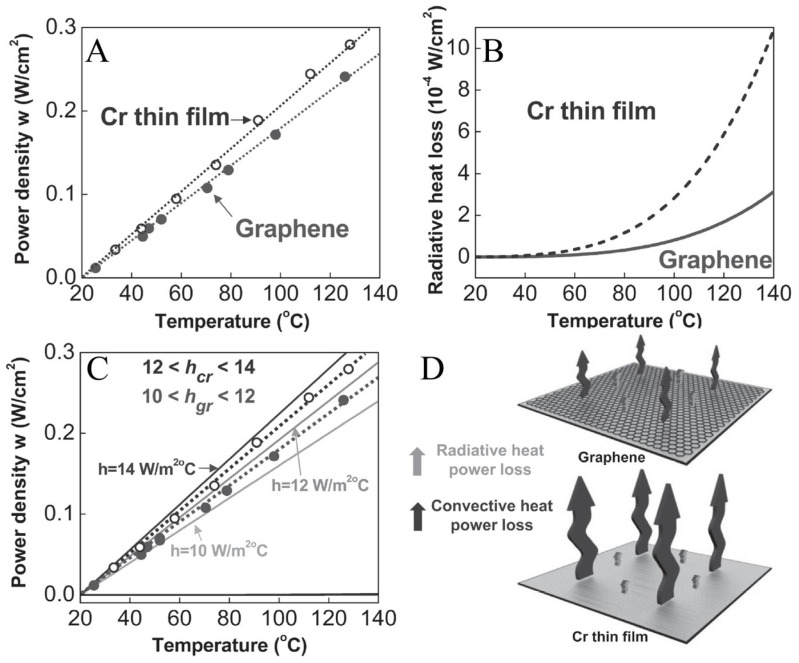
(**A**) Electrical power density vs. saturated temperature of graphene/glass and Cr thin-film/glass systems [7]. (**B**) Theoretical radiative heat power loss of two systems as a function of temperature [7]. (**C**) Convective heat power loss for various presumed convective heat-transfer coefficients with experimentally observed values for graphene (solid circles) and Cr thin-film (open circles) systems [7]. (**D**) Visualization of convective and radiative heat power loss for both systems [7].

In practical systems, the electrothermal performance of materials is influenced not only by the input power but also by heat losses associated with the material. These heat losses primarily consist of radiative and convective losses occurring at the material’s surface [119,120], as illustrated in Figure 9. Typically, the temperature of the heating system stabilizes as a result of the equilibrium between the supplied input power and the heat dissipation [7].

(3) $$mc\frac{dT(t)}{dt}=VI-(Q_{c}-Q_{r})$$

where *m, c, T*, and *t* represent mass, specific heat capacity, the temperature of the heating system, and time respectively; *V* and *I* represent input voltage and current, respectively; and *Q_c_* and *Q_r_* represent convective power loss and radiative power loss, respectively. Since the operating temperature of most electrothermal polymer composites is below approximately 100 °C, the radiative heat loss can be neglected. The convective heat power loss is expressed by the following formula:

(4) $$Q_{c}=hA(T_{m}-T_{i})$$

where *h* represents the convective heat-transfer coefficient, *A* denotes the surface area, *T_m_* represents a maximum sample temperature, and *T_i_* refers to the initial surface temperature. Therefore, at the long-term limit, the final temperature is largely determined by convective heat loss, and convective heat loss is usually related to the material. Assuming that the radiative heat power loss can be neglected compared to the convective heat power loss, the solution of Equation (3) can be obtained [34,41]:

(5) $$\frac{T_{t}-T_{i}}{(T_{m}-T_{i})A}=1-e^{-t/\tau }$$

(6) $$h=\frac{VI}{(T_{m}-T_{i})A}$$

where *T_t_* is an arbitrary temperature at time, and *τ* is the characteristic growth time constant, which indicates the temperature response time. It is obvious that the temperature change relies on the input power, convective heat-transfer coefficient, and the cross-sectional area.

From the above formula, it can be seen that *h* is directly related to *τ*. However, in previous research, the correlation was inconsistent. For instance, from the above formula and the results of Bae et al. [7], it can be seen that, in the same system, the response speed of the thermal convection coefficient *h* is inversely proportional. They discovered that the film with a lower *h* value forms a more effective thermal interface for blocking heat dissipation compared to the layer with a higher *h* value. However, this is inconsistent with the research results of Liu et al. [49], Jeong et al. [4], and Lee et al. [34]. Their studies showed that the larger the thermal convection coefficient *h*, the faster the response speed (Table 3). They found that the samples with higher *h* possessed higher improved thermal conductivity. However, in the formula, the reduction in the heating area *A* will increase the response speed, which is consistent with the result of Liu et al. [49] The results of these studies indicate that an increase in the thermal convection coefficient *h* will increase the required power density [4,7,49], as shown in Figure 9A–C. On the other hand, *h* is related to the size of the sample, the filler content in the composite material, and the type of conductive particle. As shown in Table 3, with the increase in the filler content in the composite material, *h* is increasing. Bae et al. [7] found that, among graphene, rGO, CNT, Ag, and Cr, the graphene-based system reveals the lowest convective heat-transfer coefficient due to its ideal flat surface. Furthermore, *h* is also related to the thermal resistance between the filler and the matrix. Keshtkar et al. [120] grafted polymer chains onto the surface of CNTs, and found that samples with grafted CNTs have low interface thermal resistance, thereby showing higher thermal conductivity.

## 5. The Application of Polymer-Based Electrothermal Composites

### 5.1. De-Icing/Anti-Icing

**Figure 10:**
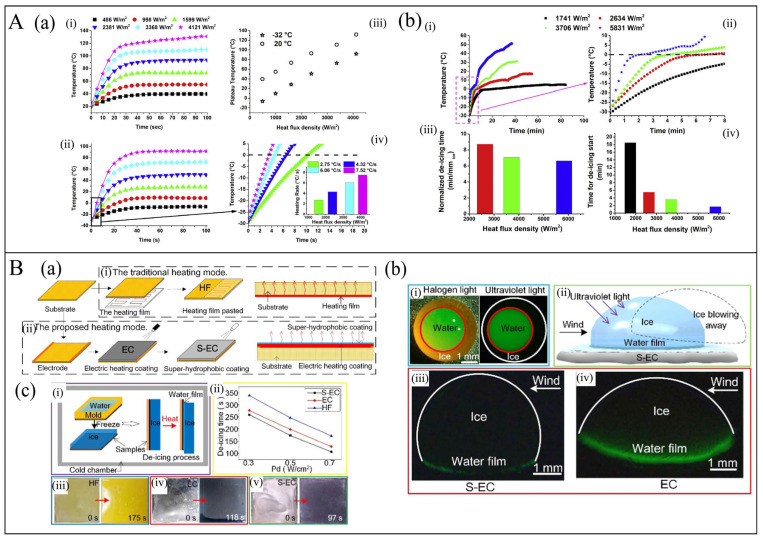
(**A**): (**a**) The Relationship between time and temperature at different heat flux densities for the film/paper at the environmental temperature of 22 °C (i), of −32 °C (ii); the relationship between heat flux densities and the equilibrium temperatures at the two analyzed environmental temperatures of 22 °C and −32 °C (iii); enlargement of the first zone of (ii) corresponding to the rectangular area highlighted with the black perimeter (iv) [44]. (**b**) (i) The relationship between time and temperature at different heat flux densities for the assembled composite during the de-icing process; (ii) increase in the temperature range from −32 °C to 10 °C; (iii) comparison of the normalized de-icing time at different heat flux densities; (iv) comparison of the response times of the assembled composite at different heat flux densities [44]. (**B**): (**a**) Schematic of the fabrication process and the heating mode of the samples: (i) The rear-mounted polyimide heating film sample (HF) and (ii) the super-hydrophobic coating combined with electric heating coating (S-EC) [14]. (**b**) Fluorescence experiment of the ice drops detaching from the coating surfaces: (i) Images of the droplet in freezing process under halogen light and ultraviolet light; (ii) the schematics of the ice drop on S-EC in this test; the momentary fluorescence images of the ice drop before it was exactly blown away on (iii) S-EC and (iv) EC [14]. (**c**) De-icing properties of the coatings: (i) The schematic of de-icing test; (ii) the de-icing time of HF, EC, and S-EC at different *P_d_*; the initial and the eventual state images of (iii) HF, (iv) EC and (v) S-EC in de-icing test at *P_d_* = 0.7 W/cm^2^ [14].

In recent years, electrothermal de-icing technology has been recognized as one of the most effective and energy-efficient de-icing methods owing to its capability for real-time online de-icing and controlling temperature. It has been widely applied in various de-icing/anti-icing applications such as aircraft [11,37,44], wind turbine blades [12,121], and roads [27,35]. Theoretically, when the temperature of the composite is heated above 0 °C, it can achieve the effect of de-icing and anti-icing. However, in practical applications, several factors must be considered, including variations in electric heating performance at low temperatures, electric heating stability, working voltage, structural integrity, and mechanical properties. Considering the structural and environmental characteristics of aircraft and wind turbine blades, most de-icing electric heating composites are designed in the form of coatings or films. Redondo et al. [12] investigated the electric heating de-icing performance of GNP/epoxy resin coatings with graphene content ranging from 8 to 12 wt.%, as well as the variation in current resistance at low temperatures. Their results demonstrated that at 800 V, the room temperature increase was between 20 and 40 °C. At low temperatures ranging from −10 to −30 °C, the resistance slightly decreased with decreasing temperature, leading to a minor increase in current. Furthermore, when graphene coatings containing 10 and 12 wt.% were sprayed onto epoxy resin used for wind turbine blades, they exhibited effective de-icing and anti-icing capabilities. To address the complex environmental conditions on aircraft surfaces and high energy consumption, Vertuccio et al. [44] integrated a flexible graphene/PVA film (mass ratio 60/40) between two carbon fiber-reinforced plastic laminates for application on the outer surface of aircraft structures. This design improved anti-icing efficiency without compromising mechanical properties or requiring the entire component to be heated. The film demonstrated excellent electrical conductivity (>1000 S/m). Comparing the temperature increase at different power densities at −32 and 20 °C (Figure 10A(a)), it was found that the temperature increase has no significant difference. Additionally, the film could heat from −32 °C to 0 °C within 10 s and remove a 1 mm-thick ice layer within 9 min (Figure 10A(b)). The de-icing time decreases with the increase in power density. Moreover, road icing in cold mountainous regions remains a common yet challenging issue. Su et al. [35] incorporated graphene into asphalt for road de-icing applications. When the graphene content was 3.0 wt.%, the resistivity of the graphene/asphalt sample was 0.87 × 10^−2^ Ω·m. At a voltage of 50 V, the temperature reached 39.3 °C. Remarkably, the addition of only 0.5% graphene was sufficient to achieve effective electric heating de-icing.

Recent studies have reported on electrothermal composites with photothermal or hydrophobic functions. These composites can significantly reduce the energy consumption for de-icing and anti-icing to a certain extent. For example, Zhao et al. [14] used the spraying method to coat MWCTN/acrylic resin and superhydrophobic SiO_2_ coatings on the glass-fiber-reinforced polymer (GFRP) substrate to prepare a super-hydrophobic coating combined with electric heating coating (named S-EC) (Figure 10B(a)). Compared with the electric heating coating (EC) and the conventional electric heating method, where the elements are embedded in fiber-reinforced plastic (HF), S-EC results in a thinner water film after heating (Figure 10B(b)). This significantly reduces the adhesion of ice due to superhydrophobicity, causing the ice to fall off as soon as it melts, ultimately reducing the energy consumption for de-icing (Figure 10B(c)), and enabling the anti-icing function without heating.

### 5.2. Multi-Functional Electrothermal Composites

**Figure 11:**
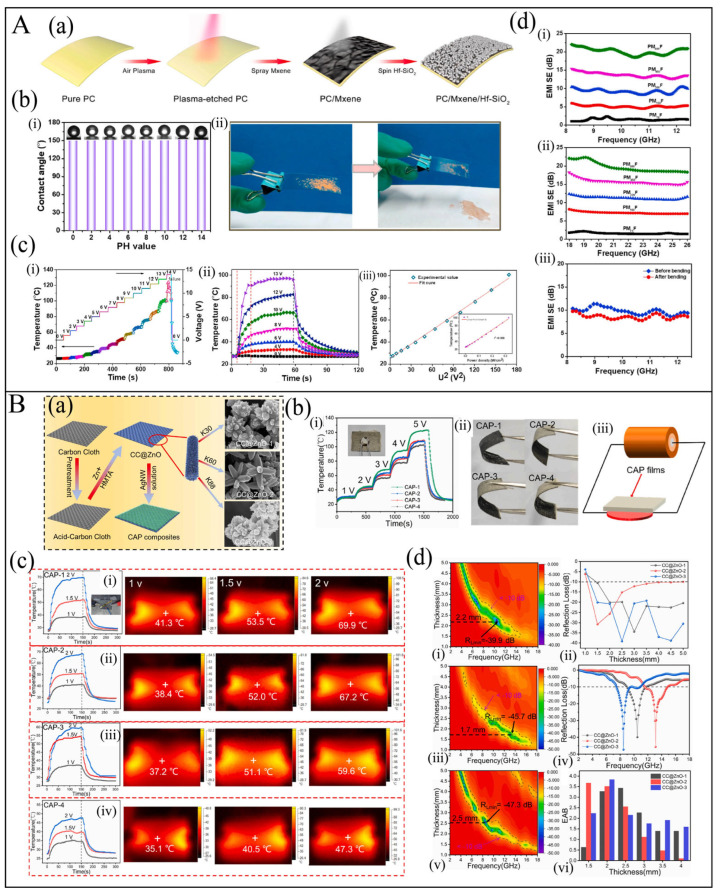
(**A**): (**a**) Schematic of the fabrication process of hydrophobic transparent PM_x_F film [122]. (**b**) (i) Contact angles (CAs) at different pH water solutions and (ii) self-cleaning process of dust on the surface of PM_x_F film [122]. (**c**) (i) Tailored surface temperatures of PM_x_F film under gradient-increasing operation voltages; (ii) the change of PM_x_F film temperature with time under different supplied voltages; (iii) linear fitting of experimental data and saturation temperature with U2 (inset: temperature versus applied power density curve) [122]. (**d**) EMI shielding performance at (i) X-band and (ii) K-band for PM_x_F film; (iii) corresponding EMI shielding performance before and after bending for 1000 cycles [122]. (**B**): (**a**) Schematic illustration of the formation process of CC@ZnO matrix and CAP composites [21]. (**b**) (i) Operating temperature vs time of ceramic heating plate; (ii) as-obtained flexible films; (iii) schematic illustration of thermal management components [21]. (**c**) Temperature curves under different operation voltages and corresponding infrared camera images of CC@ZnO/AgNWs/PVA composites with (i) 0 wt.%, (ii) 2.5 wt.%, (iii) 5 wt.%, (iv) 10 wt.% of AgNWs proportions in the solution. (**d**) (i,iii,v) R_L_ values of CC@ZnO composites with of different polyvinylpyrrolidone molecular weights; (ii) R_Lmin_ and (iv) effective absorption bandwidth values of CC@ZnO samples, (vi) the related broadband of CC@ZnO samples at different thicknesses [21].

In recent years, the single electrical heating performance has been unable to meet the application requirements. Therefore, many researchers have developed multi-functional electrical heating composites, which not only have electrical heating performance but also possess electromagnetic shielding, high thermal conductivity, absorbing waves, strain sensing, self-cleaning, etc. Electromagnetic shielding and high thermal conductivity mostly rely on the conductive network of electrical heating, while absorbing waves and self-cleaning mostly require additional second-phase or third-phase functional materials. For instance, Zhou et al. [122] orderly deposited MXene and hydrophobic fumed silica (Hf-SiO_2_) on transparent polycarbonate (PC) to develop a multi-functional film by a spraying technique coupled with spinning, as shown in Figure 11A(a). The superhydrophobic Hf-SiO_2_ coating layer not only endows PC/MXene/Hf-SiO_2_ (PM_x_F) film with excellent self-cleaning ability, but also prevents MXene from oxidation (Figure 11A(b)). Meanwhile, the film exhibits a rapid steady Joule heating performance (~100 °C at 13 V, Figure 11A(c)) and effective EMI shielding performance (SE > 20 dB) (Figure 11A(d)), simultaneously. Jia et al. [123] fabricated waterborne polyurethane (WPU)/Ag flake films by the spraying method. The WPU/Ag films show outstanding flexibility and electromagnetic interference shielding effectiveness of 68.9 dB, and can achieve a temperature of over 120 °C at 2 V. Han et al. [21] prepared carbon cloth (CC)@ZnO/silver nanowire (AgNW)/polyvinyl alcohol (PVA) (CAP) films by the scraping method (Figure 11B(a)). The film exhibited excellent thermal conductivity and outstanding Joule heating performance at low voltages (Figure 11B(b,c)). Additionally, CC@ZnO films displays high reflection loss (RL) values of −47.3 dB at 2.5 mm and a broadest effective absorption bandwidth (EAB) of 4.0 GHz in the whole X band for the construction of 3D ZnO arrays and the 2D CC matrix (Figure 11B(d)).

For multi-functional electrothermal composites, due to the addition of multiple phases in the system, the process is relatively complex. Moreover, there may be coupling phenomena among the various functions. For example, for the composite with both electric heating and electromagnetic shielding properties, while the material generates Joule heat, its various physical properties may vary with temperature increases. Further research is required to determine if the electromagnetic shielding efficiency will change due to temperature increases, and if it will be affected by the application of voltage under the voltage support.

### 5.3. Electrothermal Actuators

**Figure 12:**
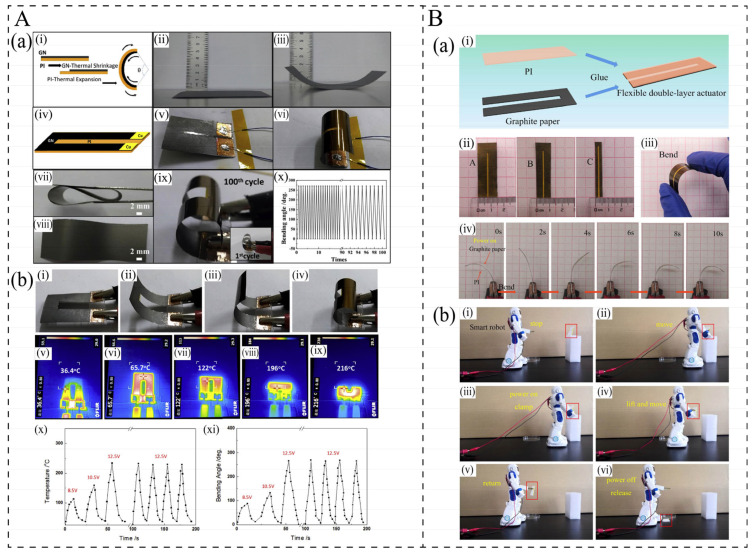
(**A**): (**a**) (i) Schematic illustration of the bending mechanism of GN/PI bi-layers due to their different thermal expansion coefficients; (ii) initial (room temperature) state and (iii) heated bending state of the GN/PI bi-layers; (iv) design structure of the GN-Fingers; (v) initial state of the GN-Fingers; (vi) bending state of the GN-Fingers under a driving voltage of 10 V, (vii,viii) Photos of the flexible GN/PI bi-layer films; (ix) photos of the soft GN-Fingers taken after the first (inset) and 100 cycles of actuation under 12.5 V; (x) bending angle of 100 cycles recorded on the as-prepared GN-Fingers [45]. (**b**) (i–iv) Images of different bending states and (v–ix) IR images of the soft GN-Fingers with the driving voltage of 12.5 V; (x) temperature of the soft GN-Finger under various driving voltages of 8.5 V, 10.5 V, and 12.5 V, also shown is four temperature cycles driven by 12.5 V; (xi) corresponding plot of the bending angle change versus time [45]. (**B**): (**a**) Diagram of the double-layer ETA: (i) Compose graphite paper and PI to fabricate flexible double-layer ETA; (ii) three specifications of graphite paper of sample A, B, C; (iii) bending the flexible actuator by hand; (iv) the bend process of actuator of sample C when power on at 6 V. [15]. (**b**) The gripping process of the smart robot: (i) stop at the initial position (ii) move to the designated location (iii) power on and clamp (iv) lift and move (v) back to the initial position (vi) power off and release [15].

In recent years, numerous researchers have applied electrothermal composites to actuators, chip storage, robotic drive arms, etc. Electrothermal actuators (ETAs), as a significant branch of electronic actuators, have garnered considerable attention [124,125]. ETAs utilize the difference in the thermal expansion coefficient of materials when Joule heat causes temperature changes to cause one side of the material to bend and thereby achieve actuation [45,126] (Figure 12A). At present, most researchers are concentrating on studying how to achieve excellent driving performance within a short period of time at low voltage. This requires the electrothermal material to have excellent electrothermal properties (high heating rate), as well as excellent flexibility, and to have a matching thermal expansion coefficient different from that of the base material. Fan et al. [15] fabricated flexible doublelayer electrothermal actuators (ETAs) made of graphite paper and polyimide (PI) film. The bending angle of the actuator can reach 248.6^◦^ and the bending curvature can reach 1.23 cm^−1^ at 6 V due to the large difference in thermal expansion coefficients between graphite and PI, and excellent electrothermal performance of graphite paper (52–360 °C) can be achieved (Figure 12B(a)). The smart gripper can grip and move objects from different directions, with excellent maneuverability (Figure 12B(b)). Wang et al. [45] successfully fabricated electrothermally driven biaxial bending artificial muscle based on an oriented graphite nanoplate nanocomposite (GN)/polyimide (PI) complex structure by a cost-effective process. GN/PI bi-layer films can be reversibly bent up to 270° at the temperature of 216 °C, with low driving voltages of 3–48 V, and the maximum bending curvature can reach 2.2 cm^−1^ (Figure 12A(b)). Secondly, some researchers have applied electric heating actuators to biosensing, diagnosis, and therapy. Kim et al. [18] prepared a soft electric heater manipulator that can lift/detach an object within 10 s and can be used repeatedly over 50 times by driving microchannels of the gel to shrink/expand, which can manipulate and transport cell/tissue sheets and ultrathin wearable biosensing devices.

## 6. Conclusions and Future Outlook

This article reviews the research progress of polymer electrothermal materials with different conductive fillers, and discusses their advantages and disadvantages. In recent years, researchers have used methods such as CVD, chemical methods, membrane transfer, self-assembly, and solution blending to prepare high-performance polymer-based electrothermal materials. For example, to address the shortcomings of traditional electrothermal materials (such as ITO and FeCr), which are prone to oxidation and have high brittleness, many researchers have utilized the optical transparency and flexibility of polymer and conductive fillers (such as CNT and graphene) to develop transparent electrothermal films. Furthermore, considering the safety of low voltages in practical applications, electrothermal composites with excellent electrical heating efficiency have been developed. For applications such as aircraft de-icing and military use, researchers have explored additional functions of electrothermal materials, such as electromagnetic shielding and high thermal conductivity, by utilizing the connected network of conductive fillers in the electrothermal materials and the functions of hydrophobicity and absorption of microwaves by introducing additional functional fillers. Using the difference in thermal expansion coefficients between conductive fillers and polymers, electrothermal drivers and artificial mechanical arms have been developed. These research efforts have led to significant breakthroughs in the study of polymer-based electrothermal composites and their application in multiple fields, including anti-fogging, anti/de-icing, electrothermal drivers, and electric heating devices for daily use.

The methods for preparing polymer-based electric heating materials have also been developed to a considerable extent; for example, CVD, the CVD + roll–roll process, solution mixing, solution mixing + spin-coating, dip-coating, the self-assembly process, spray-coating, the CVD + infiltration process, and melt blending. Among them, the solution blending method and the melt blending method are relatively simple. In particular, the melt blending method is suitable for large-scale production. However, due to the shear forces generated during melt blending that may compromise the structural integrity of other functional fillers, and the inability to produce coatings or films through this method, its application is subject to certain limitations. Furthermore, the preparation processes of some high-performance and multi-functional electrothermal materials are overly complex, with high production costs and low output, making them unsuitable for large-scale production. Therefore, from our personal point of view, simplifying the production process and increasing the output are among the development directions for polymer-based electrothermal composite materials in the future. Finally, we firmly believe that in the near future, researchers will make more breakthroughs. The practical application of polymer-based electrothermal materials in daily life and industrial fields will be further enhanced.

## Figures and Tables

**Table 3:** The thermal convection coefficient and temperature response time for the electrothermal composites.

**Composite Samples**	**Filler Content (wt.%)**	**τ** **(s)**	***h*** **(W/°C)**	**Ref**
Graphene/epoxy	2	5.67 ± 1.06	0.0017 ± 0.0005	[4]
	3	3.01 ± 0.76	0.0020 ± 0.0007	
	5	3.36 ± 0.67	0.0024 ± 0.0007	
	7	2.93 ± 0.31	0.0022 ± 0.0005	
	10	2.92 ± 0.20	0.0026 ± 0.0002	
Cr/glass	100 (glass substrate)	105	13.1 W/(m^2^·°C)	[7]
Graphene/glass	100 (glass substrate)	73	11.3 W/(m^2^·°C)	
Graphene	100	—	0.00124 W/(m^2^·°C)	
MWCNT/cellulose papers	1.5	6.1 ± 2.6	4.1 ± 1.1	[34]
	5	4.3 ± 2.2	5.6 ± 0.4	
	10.1	3.3 ± 0.5	7.2 ± 0.5	
	13.3	2.5 ± 0.5	7.9 ± 0.5	

## Data Availability

No data were used for the research described in the article.
